# Heterogeneity and renal mass biopsy: a review of its role and reliability

**DOI:** 10.7497/j.issn.2095-3941.2014.03.002

**Published:** 2014-09

**Authors:** Jeffrey J. Tomaszewski, Robert G. Uzzo, Marc C. Smaldone

**Affiliations:** ^1^Division of Urology, Department of Surgery, MD Anderson Cancer Center at Cooper, Rowan University School of Medicine, Camden, NJ, 08103, USA; ^2^Division of Urologic Oncology, Department of Surgical Oncology, Fox Chase Cancer Center-Temple University Health System, Philadelphia, PA, 19111, USA

**Keywords:** Renal cell carcinoma (RCC), renal mass biopsy (RMB), tumor heterogeneity

## Abstract

Increased abdominal imaging has led to an increase in the detection of the incidental small renal mass (SRM). With increasing recognition that the malignant potential of SRMs is heterogeneous, ranging from benign (15%-20%) to aggressive (20%), enthusiasm for more conservative management strategies in the elderly and infirmed, such as active surveillance (AS), have grown considerably. As the management of the SRM evolves to incorporate ablative techniques and AS for low risk disease, the role of renal mass biopsy (RMB) to help guide individualized therapy is evolving. Historically, the role of RMB was limited to the evaluation of suspected metastatic disease, renal abscess, or lymphoma. However, in the contemporary era, the role of biopsy has grown, most notably to identify patients who harbor benign lesions and for whom treatment, particularly the elderly or frail, may be avoided. When performing a RMB to guide initial clinical decision making for small, localized tumors, the most relevant questions are often relegated to proof of malignancy and documentation (if possible) of grade. However, significant intratumoral heterogeneity has been identified in clear cell renal cell carcinoma (ccRCC) that may lead to an underestimation of the genetic complexity of a tumor when single-biopsy procedures are used. Heterogeneous genomic landscapes and branched parallel evolution of ccRCCs with spatially separated subclones creates an illusion of clonal dominance when assessed by single biopsies and raises important questions regarding how tumors can be optimally sampled and whether future evolutionary tumor branches might be predictable and ultimately targetable. This work raises profound questions concerning the genetic landscape of cancer and how tumor heterogeneity may affect, and possibly confound, targeted diagnostic and therapeutic interventions. In this review, we discuss the current role of RMB, the implications of tumor heterogeneity on diagnostic accuracy, and highlight promising future directions.

## Introduction

The incidence of renal cell carcinoma (RCC) has been steadily increasing over the past decade^1^, in large part due to the increased incidental detection of small renal masses (SRMs) on cross-sectional abdominal imaging^2^. Nephron sparing surgery (NSS) is the standard of care for clinically localized T1a SRMs, however alternative minimally invasive and conservative treatment options are possible in select comorbid or elderly patients^3^^-^^5^. The optimal treatment modality is based on clinical assessment of patient comorbidities and tumor characteristics, but SRMs represent a heterogeneous group of benign and malignant histologic entities, with a range of clinical and biologic behaviors unpredictable by conventional imaging^6^.

Traditionally, all localized solid renal masses have been considered potentially malignant and treated with surgical excision in an effort to minimize the risk of metastatic dissemination^7^. Renal mass biopsy (RMB) has had a limited role in SRM management given concerns regarding accuracy, inconclusiveness, and complications, and its use was largely reserved for evaluation of suspected metastases or extrarenal malignancies^8^. However, given expanded treatment options including active surveillance (AS) and ablative therapies, RMB may help define tumor subtype and stratify aggressive potential, allowing for a more rational treatment protocol^7^. Indeed RMB is emerging as a safe and useful tool for the preoperative identification of benign lesions to avoid the potential morbidity of extirpative or ablative treatment, particularly in the older population^9^. However, RCC is now recognized as a heterogeneous disease process, with a number of distinct histopathological subtypes, having substantial variance in biological aggressiveness^7^. The recent identification of significant intratumoral heterogeneity in clear cell renal cell carcinoma (ccRCC) further complicates the role of RMB, as it may lead to an underestimation of the genetic complexity of a tumor when single-biopsy procedures are used^5^^,^^10^^-^^12^. Herein we review the current role of RMB, evolving indications, the implications of tumor heterogeneity on diagnostic accuracy, and highlight future directions including the promising role of RMB combined with biomarkers and molecular profiling to stratify tumor aggressiveness.

## Established indications for RMB

While extirpative therapy is the preferred management modality for clinically localized RCC^3^^,^^4^, there is emerging consensus that a significant proportion of patients with incidentally detected tumors may be over treated. Traditionally, RMB has been utilized in specific clinical scenarios in which a tissue diagnosis would obviate surgery, including lymphoma, metastatic carcinoma, infection/abscess, or concurrent with ablative therapies^7^^,^^13^. Eight percent to 13% of SRMs represent metastatic disease^14^, with lung, colon, melanoma, and liver cancer most frequently metastasizing to the kidney^15^. Among patients with a known extrarenal primary cancer, RMB has a sensitivity of 90% for malignancy detection, but over half of such lesions will prove to be new primary renal tumors^14^. Among 100 patients with non-renal malignancies diagnosed with renal masses at presentation or follow-up, progression of the non-renal malignancy and lack of enhancement of the renal mass were predictive of a metastasis to the kidney^12^.

Renal lymphoma presents as a solitary renal mass in 10% to 25% of patients, and can frequently mimic RCC on imaging^16^. Use of RMB to evaluate suspected lymphoma can establish the correct pathological diagnosis and ensure appropriate treatment with chemotherapy. A renal mass detected in the clinical setting of febrile urinary tract infection should increase suspicion for renal abscess or focal pyelonephritis and prompt percutaneous aspiration with drain placement at the time of biopsy to expedite recovery^17^. In patients with unresectable or metastatic RCC, or those who are poor operative candidates, the precise histological classification obtained from RMB can guide targeted molecular therapy^18^^,^^19^.

## Rationale for expanded RMB indications

Beyond the aforementioned established indications for RMB, concerns regarding RMB safety, diagnostic yield, accuracy, and the limited ability of RMB to influence treatment decisions based on the perception that all solid SRMs have malignant potential and should be removed with surgery upfront have limited the widespread use of RMB. However, increasing detection of incidental SRMs, development of treatment alternatives in select patients, and the discovery of several effective biologically targeted drugs for metastatic disease have raised the awareness that pretreatment tumor histology can be useful and necessary to individualize treatment decisions^6^. Increased expertise in biopsy performance and pathological interpretation of RMB, utilization of modern biopsy techniques, and increasing confidence of urologists in using biopsy results to support treatment decisions have helped to overcome the traditional limitations of RMB and fuel the renewed interest in RMB as a diagnostic tool^6^^-^^8^.

The role of RMB has expanded to include the evaluation of complex cystic lesions, SRMs <4 cm, and determination of tumor subtype (**Table 1**)^7^^,^^13^^,^^20^^-^^23^. Renal mass size is an important predictor of malignant histology, and since the odds of benign pathology significantly increase with decreasing tumor size, SRMs are benign in a significant proportion of cases^20^^,^^24^^,^^25^. In review of 2,770 solid renal mass resections over a thirty-year period, 30% of renal lesions <4 cm were benign^23^. As clinicians cannot rely on imaging alone to differentiate benign from malignant renal masses^26^, RMB can help stratify oncological risk in patients with SRM. The largest increase in incidentally detected SRMs has occurred among patients 70-89 years of age, in whom comorbidities are more frequent and the risk of competing-cause mortality is higher^27^. Competing cause mortality increases with increasing patient age, regardless of tumor size^28^, and increased comorbidity (as measured by Charlson comorbidity index) is associated with worse overall survival after surgical treatment^29^^-^^32^. The perception that active treatment for SRM may not significantly influence OS in patients with a short life expectancy has led to the development of conservative and minimally invasive treatment options for select elderly and surgically high-risk patients with a SRM^33^. For patients who are candidates for a wide range of treatment options ranging from AS to surgery, RMB can be useful in the management of all solid, contrast-enhancing SRMs when histologic diagnosis has the potential for supporting treatment decisions^7^. Among young and healthy patients, RMB is not routinely recommended because long-term oncologic outcomes of non-surgical therapies are not available, and there may be a risk of histologic transformation when a renal tumor is observed for a prolonged period of time^6^.

**Table 1:** Current indications and contraindications for renal mass biopsy

Indications
Absolute
Indeterminate SRM on abdominal imaging
Suspicious renal mass and known extrarenal malignancy
Incidentaloma in candidates for AS or ablative therapy
Suspected lymphoma
Confirm histologic success and monitor for recurrence following thermal ablation
Renal mass and febrile UTI, possible abscess
Metastatic renal tumor, to select optimal biologic systemic therapy
Unresectable retroperitoneal tumors involving the kidney
Relative
Uni-/bilateral multifocal tumors
Solitary kidney
Medically unfit
Emerging
Enhancing SRM
Indeterminate cystic lesions
Determine histologic subtype in metastatic RCC
Contraindications
Coagulopathy (uncorrected)
Patients who are not candidates for any type of therapy (surgery, ablation, medical therapy) given limited life expectancy

An exciting and expanding indication for RMB is assessment of renal primary lesions in patients with metastatic RCC. Over the past decade, the treatment landscape in metastatic RCC has changed dramatically^31^, and identification of histologic subtype and relevant molecular pathways may allow for more precise targeted systemic treatment^32^^,^^34^^-^^36^. Identification of sarcomatoid differentiation, for example, represents a poor prognosis with limited response to systemic treatment and may represent a contraindication to cytoreductive nephrectomy to avoid morbidity^6^^,^^37^. Clinical responses to sunitinib and sorafenib are low in papillary RCC^35^, but efficacy of the mTOR inhibitor temsirolimus appears more pronounced in non-clear cell and papillary type RCC^36^. RMB of the primary renal tumor allows ideal targeted therapy selection, and is recommended when a cytoreductive nephrectomy is not indicated or when neoadjuvant systemic therapy is planned^4^. In the metastatic RCC setting, interest in precision beyond “cancer” versus “benign” can be challenging, reflecting increased tumor heterogeneity, occasional divergent pathologies, and predominance of tumor necrosis^7^^,^^14^^,^^38^.

## Clinical nomograms to predict malignant potential

Efforts have also been focused on development of clinical nomograms to predict malignant potential prior to surgery and safely substitute for RMB. Early efforts to predict malignant pathology and tumor grade using tumor size and other clinical variables (such as age, gender, smoking history and presence of symptoms) were highly inaccurate, which limited their clinical utility^39^^-^^41^. Combining individual descriptors of the nephrometry score with patient characteristics (age, gender), Kutikov *et al*.^41^ developed a nomogram that could predict malignant RCC histology and high-grade features. Recently externally validated^42^, these models represent the most accurate preoperative predictors of malignant potential of localized renal tumors to date, and their accuracy for predicting tumor grade matches that of percutaneous core biopsy^40^. Although early efforts have been encouraging, the role of statistical modeling, including nomogram development, for risk prediction during AS is likely to evolve and expand in the future^43^.

## Safety

RMB is a relatively safe procedure with minimal morbidity. Contemporary series reveal overall complications rates ranging from 1.4%^7^^,^^44^^-^^51^ to 4.7%^13^^,^^14^^,^^52^^-^^57^, with major complications reported in 0.46%^7^^,^^13^^,^^44^^,^^45^^,^^47^^,^^48^^,^^58^^,^^59^. Potential complications of RMB include bleeding, tumor seeding, infection, pneumothorax, and arteriovenous fistula^6^^,^^7^^,^^56^. Most RMB related complications are minor and related to bleeding, but clinically significant bleeding is unusual and almost always self-limiting. While small pneumothoraces can occur, especially following biopsy of posterior upper pole tumors, they are rare and usually managed conservatively^48^. The most feared and controversial potential complication of RMB is tumor seeding of the biopsy tract. The overall estimated risk of tract seeding is <0.01%^6^^,^^11^^,^^60^^,^^61^ with only a handful of case reports documenting its occurrence and one reported case since 1994^62^, tumor seeding should be considered anecdotal^21^. Among 1,377 patients undergoing RMB in contemporary series using coaxial techniques with guides or cannulas, no cases of biopsy tract tumor seeding were reported^6^. While the risk of RMB related complications is small but not zero, risks should be weighed against managing a patient with suboptimal information.

## Diagnostic accuracy for malignancy

The ultimate goal of RMB is to appropriately match tumor treatment with tumor biology, so its utility becomes dependent upon the clinical scenario encountered and the accuracy of RMB for determination of malignancy and tumor grade. The diagnostic accuracy of RMB is currently estimated at >95%^7^, but care must be taken when determining accuracy because the definitions used vary and can result in artifactually high estimates. Most series define accuracy as the percentage of informative biopsies for which the pathological diagnosis appeared to be correct; that is not a false-positive or a false-negative, based on final surgical pathology or radiographic and clinical surveillance^7^. This definition is inherently limited, however, as it assumes that radiographic surveillance is a reliable surrogate for pathologic diagnosis, and ignores non-informative biopsies.

Historically, many non-informative biopsies were inappropriately considered “false-negative”, representing a major criticism of RMB, in that missed malignancies would potentially remain untreated^7^. Among recent series, diagnostic yield of RMB ranges from 78% to 100%, while sensitivity and specificity for the diagnosis of malignancy are 86%-100% and 100%, respectively (**Table 2**)^9^^,^^44^^,^^45^^,^^48^^,^^49^^,^^51^^,^^54^^,^^59^^,^^61^. Among review of 2,474 recent RMB results, PPV and NPV for the diagnosis of malignancy were 97.5% and 82%, respectively, with an overall sensitivity of 92.1% and specificity of 89.7%^13^. While the rate of non-diagnostic biopsy remains in the range of 10%-20%, in patients with an initially non-diagnostic biopsy, the diagnostic rate on re-biopsy ranges from 75% to 100%^44^^,^^47^^,^^49^^-^^52^^,^^54^^,^^58^^,^^59^^,^^65^^-^^68^. Although tumor size, location, and character can certainly play a role^9^, the similarity between re-biopsy and initial biopsy rates suggests there is nothing intrinsic to tumors themselves that results in a nondiagnostic biopsy and that repeat biopsy is both feasible and can be expected to identify tumors and cancers^9^. It is important to note that a nondiagnostic biopsy is not a surrogate for a benign diagnosis and should not be considered a reassurance that the patient has a benign lesion^9^.

**Table 2:** Contemporary outcomes from renal mass biopsy series

Series	Cases	Accuracy (%)				Complications (%)
		Diagnosis	Malignancy	RCC subtype	Grade	
Veltri *et al*.^61^	103	100	NA	93.2	NA	5.3
Dechet *et al*.^63^	100	100	76	NA	NA	NA
Richter *et al*.^64^	205	62.4	38.3	NA	NA	NA
Lebret *et al*.^59^	119	79	86	86	74*	0
Maturen *et al*.^45^	152	96	Sensitivity 97.7; specificity 100	NA	NA	1.3
Shannon *et al*.^44^	235	78	100	98	NA	0.9
Volpe *et al*.^49^	100	84	100	100	75*	1
Leveridge *et al*.^9^	345	80.6	99.7	88	63.5	0.3
Veltri *et al*.^61^	150	100	NA	93.2	NR	0
Wang *et al*.^51^	110	90.9	100	96.6	NR	1.8

RCC, renal cell carcinoma; *, classified as low (Fuhrman grade I/II) or high (Fuhrman grade III/IV). Restricted to series with at least 100 biopsies performed for brevity.

## Inaccurate RMB

Inaccurate RMB, including true false-negative and false-positive results, represents the most concerning outcome for clinicians. Concern is justified, as false-negative results could lead to surveillance of a malignancy with metastatic potential. Fortunately, the rate of false-negative RMB (excluding non-informative RMB) among modern series ranges from 0% to 3.8%^7^^,^^13^^,^^48^^,^^51^^,^^69^^,^^70^. Sampling error, tumor necrosis, and tumor heterogeneity are responsible for most false-negative biopsy results^14^. Smaller tumors can be more difficult to visualize and target^56^, but larger tumors are also prone to sampling error given the greater incidence of necrosis^14^^,^^65^. In a series of 115 core RMBs, the false-negative rate was lowest for tumors 4 to 6 cm in diameter (2.3%), compared to small (1 to 3 cm; 13%) and large (>6 cm; 12%) tumors^14^. In a larger series of 345 RMBs, the odds ratio for a diagnostic result was 2.3 (95% CI, 1.5-6.3) for each 1-cm increase in tumor size^9^.

Concern for a coexisting malignancy in otherwise benign tumors is also a significant barrier to routine RMB, undermining the validity of RMB and likely deterring its routine use^13^^,^^67^. While hybrid histology has been largely described in patients with multifocal tumors and known genetic syndromes, concern persists despite relatively sparse data on sporadic solitary tumors^68^. In the largest series to date to examine the rates of coexisting malignant and high grade pathology, 1,829 patients with benign solid solitary renal tumors underwent tumor excision, and 147 masses were found to contain a benign component [oncocytoma (5.2%), angiomyolipoma (2.4%), or another solid benign pathology (0.38%)]^68^. Only four patients (2.7%) had hybrid malignant pathology, all of which were chromophobe in the setting of oncocytoma, and importantly, no benign tumor coexisted with high grade malignancy^68^. Hybrid tumor rates as high as 27.1% have been reported^67^, but this discrepancy stems from the pathological criteria used to classify the malignant component, and hybrid tumors are generally believed to be nonaggressive^67^^,^^68^^,^^71^^,^^72^. Collectively, these data suggest that uncertainty regarding hybrid malignant pathology coexisting with benign pathological components should not deter renal biopsy in efforts to minimize over-treatment of the renal mass, especially in the frail and comorbid populations^68^.

## Accuracy of tumor grading and histological subtype

**Figure 1:**
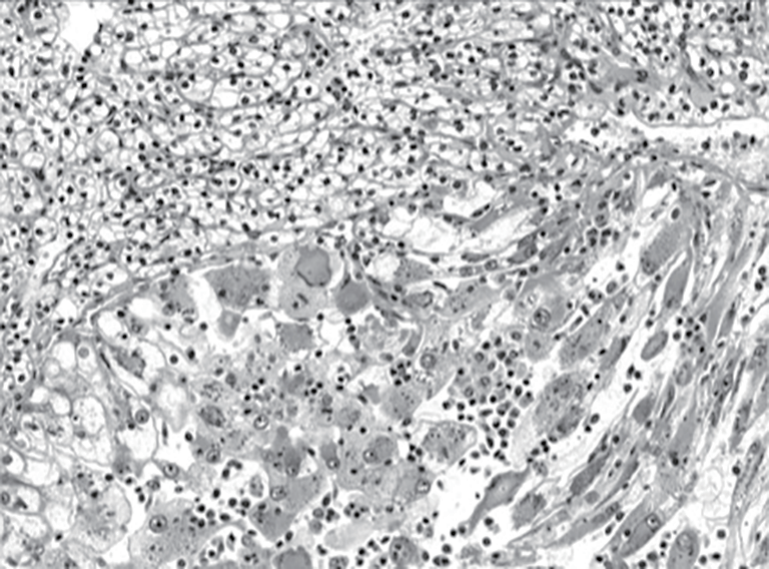
Photomicrograph from nephrectomy specimen illustrates intratumor grade heterogeneity. Low grade tumor cells with Fuhrman grades 1 and 2 nuclei (upper half) are sharply demarcated from high grade tumor cells with Fuhrman 3 and 4 nuclei (lower half). In addition, many high grade tumor cells had rhabdoid features, characterized by densely eosinophilic cytoplasmic aggregates of intermediate filaments (reproduced with permission from Elsevier)^56^.

Histological subtype and grade are known prognostic factors in renal carcinoma and potentially important in the staging and management of small renal cancers^26^. The accuracy of grading renal cell cancers with percutaneous biopsy is controversial and largely unreliable, with reported accuracy for grading ranging from 43% to 75%^26^^,^^49^^,^^54^^,^^59^^,^^70^^,^^71^. Even the most recent series reveal only moderate concordance between biopsy and surgery grade (Kappa score 0.52)^26^. By compressing the Fuhrman nuclear classifications, accuracy of differentiation between “low” and “high” grade tumors can improve to as high as 93%^26^. The reproducibility of these findings will remain in question however until verified by additional larger reports. Fuhrman grade intratumor heterogeneity further complicates accurate grade determination via RMB, and grade heterogeneity in a single tumor has been observed in up to 25% of cases (**Figure 1**)^72^. While not as powerful a prognostic factor in RCC nomograms and predictive models^73^, diagnostic accuracy for histologic subtyping has also been examined. When core RMBs are compared to nephrectomy specimens, accuracy for subtyping is high, ranging from 86% to 100%^26^^,^^44^^,^^51^^,^^56^^,^^59^. Given patients with clear cell RCC have a poor prognosis compared to those with papillary Type 1 and chromophobe RCC^74^, accurate subtype determination may impact clinical management.

## Increasing the accuracy of conventional RMB

Utilization of molecular characteristics and refined RMB techniques has the potential to improve the accuracy of conventional RMB. Use of larger (18 gauge) needles is safe and allows acquisition of sufficient tissue for accurate diagnosis and may increase the diagnostic accuracy of RMB^56^. The presence of sarcomatoid dedifferentiation or necrosis correlates with decreased recurrence-free survival^75^^-^^78^, and expression of carbonic anhydrase IX, a ccRCC marker, is an independent predictor of survival^79^. Adding fluorescence *in situ* hybridization to evaluate chromosomal abnormalities increased the diagnostic accuracy of *ex vivo* RMB from 87% with histopathology alone to 94%^77^, and addition of real-time polymerase chain reaction data on the expression of 4 select genes showed similar promise^78^. In evaluation of RMB tissue from 60 tumors, use of molecular diagnostics increased the accuracy of histological subtyping form 90% to 95%^78^. Microarray technology has demonstrated some ability to differentiate tumors by gene expression profiling^80^, and the molecular fingerprinting of histological subtype could someday be used to predict likelihood of recurrence^13^. Gene expression microarrays have been used to classify aggressive variants of RCC^81^, and these profiles are concordant with final surgical pathology^80^. Combining molecular profiling with patient factors, tumor size, and radiographic parameters may provide refined risk-stratification of patients with renal tumors for counseling and management.

## Intratumoral heterogeneity

A potential limitation in the imagined future of oncology is its underestimation of tumor heterogeneity—not just heterogeneity between tumors, which is a central feature of the new image of personalized medicine, but heterogeneity within an individual tumor (**Figure 1**)^82^. Gerlinger *et al*.^11^ performed unbiased whole-exome sequencing of multiple primary and metastatic renal-cell carcinoma tumor sites in several different patients to map genetic heterogeneity within a single tumor. A majority of somatic genetic mutations were not present ubiquitously within a tumor, and branched evolutionary tumor development was evident^11^. Approximately two thirds of the mutations (including mutations, allelic imbalance, and ploidy) that were found in single biopsies were not uniformly detectable throughout all the sampled regions of the same patient’s tumor^11^. “Favorable” and “unfavorable” prognostic gene profiles were expressed in different regions of the same tumor. Unlike previous studies utilizing next-generation sequencing of a single index lesion per patient and targeted sequencing of the mutated genes in other sites, the author’s independently sequenced and validated mutant gene expression and altered function throughout primary and metastatic sites.

Further, there were widespread alterations in the total number of tumor cell chromosomes (aneuploidy) and detection of many allelic imbalances at the chromosomal level, in which one allele of a gene pair is lost^11^^,^^12^^,^^82^^,^^83^. These imbalances can be due to chromosome loss or gene imprinting and may alter gene expression^82^. Convergent evolution was also evident, with different tumor regions containing different mutations within the same genes. This underscores the importance of changing particular tumor-cell functions as the tumor expands and evolves^11^^,^^12^^,^^82^^,^^83^. Tumor heterogeneity presents a considerable therapeutic challenge because treatment choices based on a biomarker present in a single biopsy specimen may not be relevant^72^, and genomics analyses from single tumor-biopsy specimens may underestimate the mutational burden of heterogeneous tumors^11^. Thus, a single tumor biopsy, the standard of tumor diagnosis and the cornerstone of personalized-medicine decisions, cannot be considered representative of the landscape of genomic abnormalities in a tumor. Given that selective gene activation and inactivation occurs to guarantee tumor survival, the genes that are affected by convergent evolution may be suitable targets for functional inhibition or restoration. However, the concept of directing therapy on the basis of genetic tumor markers is probably too simple. Reconstructing tumor clonal architectures and the identification of common mutations located in the trunk of the phylogenetic tree may contribute to more robust biomarkers and therapeutic approaches^11^.

## Risk stratified RMB and utility in complex scenarios

**Figure 2:**
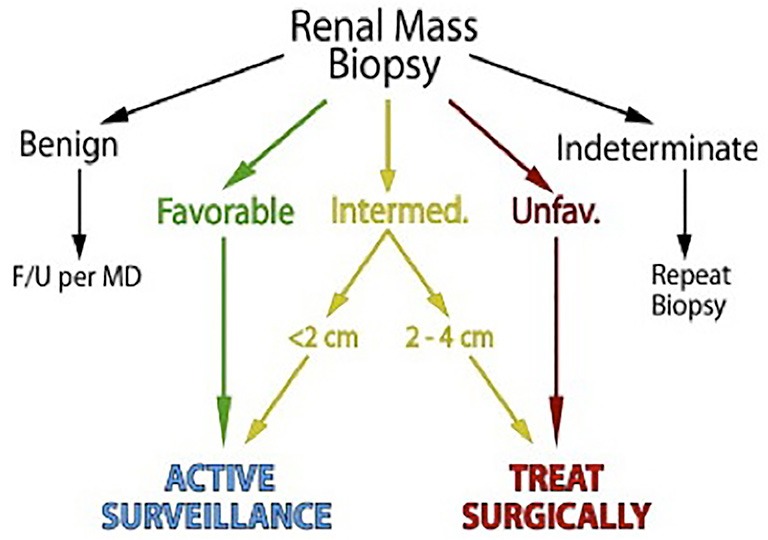
Simplified biopsy directed management algorithm designating active surveillance *vs*. treatment based on mass size and histological risk category (reproduced with permission from Elsevier)^72^.

Incorporation of RMB results could allow clinicians to reduce the treatment burden for patients without compromising disease specific survival, and incorporation of RMB into risk stratified management algorithms has been proposed as the next refinement in RMB^72^. Using management based on final pathology as the reference standard for patients with SRM ≤4 cm, RMB had a 100% treatment PPV and 69% surveillance NPV for correctly determining management^72^. Revision of the histological risk-grouping to account for undergrading of grade 1 ccRCC on biopsy increased sensitivity to 96% and improved the negative (surveillance) predictive value to 86%, while the positive (treatment) predictive value remained at 100%^72^. Using risk stratified histology groups and maximum mass diameter on imaging accurately defines patients as surveillance or treatment candidates, and incorporation of additional factors could improve the results. Although silent discrepancies may exist between initial biopsy and final pathology due to the heterogeneous nature of some tumors, this variability becomes clinically irrelevant when using a well-defined management protocol^72^. A “biopsy for all” strategy might avoid unnecessary surgeries, even among young patients with an incidentaloma (**Figure 2**).

Patients with bilateral synchronous (BSRT) or unilateral multifocal renal tumors pose multiple prognostic and therapeutic challenges, similar to those of patients with hereditary RCC^84^^-^^87^. Numerous issues need to be addressed in patients with BSRT, including the need for complex nephron-sparing surgery, staged procedures, prognosis, decline in renal function, the prospect of progressive renal insufficiency, hyperfiltration injury, and the need for future renal replacement therapy^85^. Malignant and nuclear grade concordance rates are high for contralateral disease, ranging from 84%-95% and 79%-85%, respectively, while benign concordance rates are much lower (39%-67%)^85^. The data suggest that RCC of any type present on one side indicates RCC will be present on the contralateral side, but when benign disease is present, there is a lower risk of only benign disease on the contralateral side^85^. In patients with BSRT and confirmed benign disease on one side, consideration of contralateral RMB is reasonable.

Between 5% and 25% of patients who undergo surgery for a presumed single renal mass are found to have multifocal disease^83^, and diagnosis of unilateral synchronous multifocal renal masses at presentation impacts the intensity of evaluation, use of RMB, and treatment planning. Although the pathological concordance of sporadic bilateral masses is relatively high, rates of pathological concordance from unilateral multifocal cases of RCC are poorly defined. In the largest series to date, 97 patients with unilateral synchronous multifocal renal masses underwent partial nephrectomy^84^. Malignant, benign, histological concordance rates were 77.2%, 48.6%, and 58.8%, respectively^84^. The low concordance rates indicate that single renal mass biopsy in patients with multifocal disease may be insufficient patient counseling and treatment planning.

In patients presenting with a renal tumor and clinical evidence of metastatic disease, RMB of the primary tumor and/or metastatic lesion can be done to obtain a tissue diagnosis. Indications for primary tumor RMB include inability to make a tissue diagnosis from a metastatic site, atypical appearance of the primary tumor on preoperative imaging, suspicion of multiple primary neoplasms and/or the need to make a histological diagnosis to guide treatment^86^. In the largest series evaluating percutaneous RMB findings compared to nephrectomy specimens in patient with metastatic RCC^86^, tumor grade was accurately assessed in only 33% of cases and discordance by two or more grades was reported in 17%^86^. Sarcomatoid dedifferentiation was found in 20.5% of final pathological specimens and yet preoperative biopsy failed to identify this in almost 90%. Biopsy failed to specify the primary histological subtype in 41% of cases and many biopsies were non-diagnostic for RCC^86^. Physicians should use caution when using biopsy data assigning grade or sarcomatoid elements to enroll patients with metastatic RCC in neoadjuvant clinical trials or to make complicated treatment decisions. Future development of better imaging techniques, new molecular markers and/or improved immunohistochemical techniques may help improve the predictive accuracy of percutaneous biopsy of large heterogeneous primary tumors in patients with metastatic RCC^86^.

## Future directions

The addition of molecular and genetic tests on RMB specimens has exciting potential to provide prognostic information useful for treatment decisions^6^. Markers associated with renal cell carcinogenesis and progression (VHL, HIF-1α, VEGF, CAIX, pS6, phosphatase and tensin homolog) and markers described in other malignancies (p53, Ki67, matrix metalloproteinases 2 and 9, IGF II mRNA binding protein, and survivin) are currently under investigation^87^. In an analysis of 170 patients who underwent nephrectomy for RCC, markers related to the HIF and mTOR pathways were analyzed, and the expression of Ki-67, p53, endothelial VEGF receptor 1, epithelial VEGFR-1, and epithelial VEGF-D were independent predictors of disease-free survival^88^. A nomogram combining the aforementioned molecular markers with clinical and pathological variables yielded a prognostic accuracy of 90%^88^.

Cytogenetic alterations in clear cell RCC, such as loss of 9p, are known to correlate with a significantly worse 5-year cancer-specific survival^89^, and have been examined among a cohort of 282 patients^90^. Combining loss of 9p (as measured by FISH) with clinical stage and Fuhrman grade into a nomogram yielded high prognostic accuracy to predict 3-year cancer-specific survival^90^. DNA or RNA expression microarrays can also be performed using core biopsy specimens, although adequate tissue is needed to effectively extract DNA and RNA for analysis. One recently identified gene array was able to distinguish two groups of clear cell RCC’s with significantly different 5-year recurrence-free survival rates of 68% and 42%, respectively^64^^,^^80^^,^^84^^,^^91^^,^^92^. While results of early genetic and molecular tissue markers are highly promising, prospective studies are needed to validate the findings and expand applicability to non-clear cell histotypes.

## Limitations

While the current renaissance in RMB is exciting, several notable limitations deserve mention. A definite assessment of RMB accuracy remains difficult given most series are small, single institutional, and use varying definitions for biopsy success^6^. The accuracy of RMB is limited by factors intrinsic to the procedure (inconsistent tumor sampling), to the histology of renal tumors (difficult differential diagnosis of tumor histotypes, evaluation of Fuhrman grade, presence of intratumoral heterogeneity), and to the interpretation of biopsy specimens (interobserver variability in pathologic assessment)^6^. Sampling error and tumor heterogeneity contribute to inaccuracy of RMB, and differentiation among conventional RCC with granular cytoplasm, oncocytic papillary RCC, the eosinophilic variant of chromophobe RCC and oncocytoma can also be particularly problematic^13^. Further practical concerns include the risk of renal hemorrhage given renal vascularity, and technical failure leading to indeterminate or inaccurate pathological findings. Finally, assessment of Fuhrman grade on RMB is challenging with suboptimal accuracy^9^^,^^15^^,^^48^^,^^49^^,^^51^^,^^52^^,^^59^^,^^66^, moderate interobserver concordance for grade assessment, and intratumoral grade heterogeneity in 5%-25% of renal tumors^48^.

## Conclusion

Advances in the understanding of the limited biological potential of many SMRs, expanding treatment and surveillance options for RCC, improved biopsy techniques, and the integration of molecular factors into prognostic and therapeutic algorithms have renewed interest in RMB. Current indications for RMB include diagnostic work-up of renal tumors that are indeterminate on imaging, assessment of the primary renal tumor prior to initiation of systemic therapy for metastatic RCC, and of incidentally detected radiologically suspicious SRMs in patients at high surgical risk to support treatment decisions and avoid unnecessary surgery. Intratumoral heterogeneity, sampling error, and inconsistent classification of RMB failures in published studies make a precise determination of RMB accuracy difficult. Uniform reporting of RMB safety and efficacy in the literature as well as further studies addressing tumor heterogeneity and sampling error are needed. Differentiation of oncocytoma from oncocytic neoplasms poses a diagnostic dilemma, but incorporation of more sophisticated molecular analyses into enhanced RMB has promising potential.

Despite these limitations, RMB has a definite and expanding role in the evaluation and treatment of renal masses, but remains significantly underutilized. While the ability to differentiate between high and low grade malignancies remains the chief limitation of RMB, we anticipate the further integration of percutaneous biopsy into clinical algorithms will guide patient counseling and inform personalized decision making. Future studies will focus on the role of repeat biopsy and the use of biomarkers and molecular fingerprinting in order to facilitate a more rational approach to the management of renal masses.
